# A prospective study of the association of weekend catch-up sleep and sleep duration with mortality in middle-aged adults

**DOI:** 10.1007/s41105-023-00460-6

**Published:** 2023-05-05

**Authors:** Takuya Yoshiike, Aoi Kawamura, Tomohiro Utsumi, Kentaro Matsui, Kenichi Kuriyama

**Affiliations:** 1 Department of Sleep-Wake Disorders, National Institute of Mental Health, National Center of Neurology and Psychiatry, 4-1-1 Ogawahigashi, Kodaira, Tokyo 187-8553 Japan; 2 Department of Laboratory Medicine, National Center Hospital, National Center of Neurology and Psychiatry, Kodaira, Japan

**Keywords:** Middle-aged adults, Mortality, Polysomnography, Sleep debt, Sleep duration, Weekend catch-up sleep

## Abstract

Health effects of weekend catch-up sleep (CUS) could differ depending on both the ability to obtain sufficient sleep during weekdays and amount of weekend CUS required to compensate for sleep lost during the week. ﻿Using data from 3128 middle-aged (40–64 years) participants of the Sleep Heart Health Study, we examined the longitudinal association of these two aspects of sleep with all-cause mortality. CUS was calculated as the difference in self-reported habitual sleep duration between weekends and weekdays, and classified into no, short (1 h), and long (2 h or more) CUS. Polysomnography-measured total sleep time, representing the ability to obtain sufficient sleep, was classified into short (< 360 min) or normal (≥ 360 min) sleep durations. We estimated multivariable-adjusted mortality hazard ratios (HRs) and 95% confidence intervals (CIs) for six groups divided by the extent of CUS and sleep duration. Participants were followed up for a median (interquartile range) of 12.3 (11.3–13.5) years. Short weekend CUS with normal sleep duration was associated with lower mortality compared to no CUS with normal sleep duration (﻿HR, 0.48; 95% CI 0.27–0.83). When stricter cutoffs were applied for sleep durations, while the protective effect of short CUS with normal sleep duration (≥ 390 min) was strengthened (HR, 0.36; 95% CI 0.17–0.78), the harmful effect of short CUS with short sleep duration (< 330 min) emerged (HR, 1.84; 95% CI 1.08–3.14). Results highlight the importance of balancing sleep ability and CUS. Sufficient sleep may reduce weekday sleep debt and only a short CUS would be required on weekends, improving mortality in middle-aged adults.

**Supplementary Information:**

The online version contains supplementary material available at 10.1007/s41105-023-00460-6.

## Introduction

Insufficient sleep has been recognized as a risk factor for human health outcomes, including obesity, diabetes, hypertension, cardiovascular disease, cognitive decline, and all-cause mortality [1, 2]. However, consistently sleeping as long as needed is still a challenge for many of us, because we often voluntarily curtail sleep to maximize time for daily social activities. Nightly sleep loss accumulates over days, resulting in a buildup of chronic sleep loss (sleep debt). Although extending sleep, or catch-up sleep (CUS), on weekends is a common way of making up for sleep lost during the week, it is currently debated whether weekend CUS could cancel out some of the health risks associated with sleep loss and circadian misalignment [3–5].

Experimental studies have shown that weekend CUS could not make up for even one hour of nightly sleep lost during weekdays [6, 7]. On the contrary, an epidemiological study has suggested that weekend compensatory sleep mitigates the harmful effect of short sleep during weekdays on all-cause mortality [4]. These mixed findings suggest that the health effects of weekend compensatory sleep could differ depending on both the amount of CUS and one’s ability to obtain sufficient sleep. The ability to obtain sufficient sleep could be generated under the control of homeostatic and circadian drives, both of which are interfered with by aging, social constraints, and/or certain pathologies [8]. Following the homeostatic regulation of sleep, it is plausible that the greater the weekday sleep reduction, the greater the amount of weekend CUS, reflecting sleep debt. Therefore, there may be a threshold value for lost sleep during weekdays, below which weekend CUS could successfully pay off the sleep debt. Additionally, the ability to obtain a certain amount of sleep and the sufficient opportunity to sleep during weekdays may be vital to minimize sleep debt. Obtaining sufficient sleep during weekdays decreases the degree to which CUS is required on weekends. Moreover, consistent with the idea that almost everyone is constantly sleep deprived ﻿and carries some amount of sleep debt [7, 9, 10], weekend sleep extension may have a favorable effect [7]. Nonetheless, this possible favorable effect is more likely to emerge among individuals who maintain their ability to obtain sufficient sleep and need only a small amount of weekend CUS within their controllable range, than among those who are impaired in their ability to obtain sufficient sleep or need a greater amount of weekend CUS beyond their controllable range. As with other physiological disciplines, an objective measurement of sleep duration (e.g., polysomnography [PSG]), compared to self-reports of sleep duration, could allow for a more reliable assessment of the ability to obtain sufficient sleep. Although sleep experts recommend sufficient sleep duration for seven to nine hours per day for young to middle-aged adults [11], a considerable mismatch between self-reported and objectively measured sleep duration has been found across studies, with the former being longer on average than the latter [1, 12]. This subjective–objective sleep discrepancy is likely, at least partly, to result from a general tendency to overestimate one’s sleep duration subjectively^11^. We investigated the combined effects of the degree of weekend CUS and PSG-measured sleep duration on long-term mortality outcomes in middle-aged (40–64 years) adults ﻿using data from the Sleep Heart Health Study (SHHS), a multicenter population-based prospective cohort study [13, 14].

## Methods

### Participants

All data were derived from the SHHS and other details of the study are available in previously published literature [13]. The study was performed in accordance with the Helsinki Declaration, and each participant provided written informed consent. A total of 6441 participants aged 40 years and older were enrolled from existing cohorts and underwent the baseline examination between 1995 and 1998. Of these, 3128 middle-aged (40–64 years) participants who underwent overnight PSG were included in the current investigation. Across community-based cohorts, including the SHHS, older adults less frequently sleep longer on weekends than on weekdays [1, 4, 15, 16]. Therefore, we only included middle-aged adults in our analysis. The distinction between middle-aged and older adults relied on the National Sleep Foundation’s expert consensus age categories [17]. The current project was approved in April 2020 by the Ethics Committee of the National Center of Neurology and Psychiatry (project number: A2020-012). All analyzed data are publicly available (sleepdata.org). This study is reported following the Strengthening the Reporting of Observational Studies in Epidemiology (STROBE) reporting guideline for cohort studies.

### Measures

#### Objective sleep measure

We employed participants’ PSG-measured total sleep time (TST) as an objective index of the ability to maintain sleep. An unattended, portable in-home PSG was conducted during the baseline examination, using the Compumedics P Series System (Abbotsford, Victoria, Australia). The vast majority of participants underwent in-home PSG on a weekday (*n* = 2866, 91.6%). Standard PSG characteristics, including TST (total time in non-rapid eye-movement stages 1–3 and rapid eye movement sleep), were evaluated based on the SHHS Reading Center manual of operations, as described previously [18].

#### Subjective sleep measure

During the baseline examination, the participants were asked to report their habitual sleep duration at night on weekdays (or workdays) and on weekends (or non-workdays) in hours.

### Primary exposure

The primary exposures were a specific amount of habitual weekend extra sleep (short CUS, or long CUS vs. no CUS) and short sleep duration determined on a PSG night (vs. normal sleep duration), both of which were obtained at the baseline examination. The primary definition of short sleep duration was TST of less than 360 min (6 h) on PSG, a cutoff commonly used to define objectively short sleep among adults in epidemiological studies, including the SHHS [19–21]. Additionally, we applied two stricter cutoffs for short and normal sleep durations (< 330 min, and ≥ 390 min, respectively) to explain how impaired or maintained sleep affects the associations between weekend CUS and mortality. The amount of weekend CUS was calculated as the difference in habitual sleep duration between weekends and weekdays, and classified into no CUS, short (1-h) CUS, and long (2-h or more) CUS, based on previous studies [22, 23]. The two TST and three CUS classifications were combined to generate six TST-CUS classifications, i.e., no CUS with short TST, short CUS with short TST, long CUS with short TST, no CUS with normal TST, short CUS with normal TST, and long CUS with normal TST.

### Mortality outcome

Deaths from any cause were identified using multiple concurrent approaches, including follow-up interviews, written annual questionnaires or telephonic conversations with participants or their next-of-kin, surveillance of local hospital records and community obituaries, and linkage with the Social Security Administration Death Master File, as described elsewhere [24].

### Other covariates

Baseline sociodemographic and health covariates included age, sex, race/ethnicity (Caucasian and other), smoking status (current, former, and never), body mass index, hypertension (defined as an average systolic blood pressure > 140 mm Hg or average diastolic blood pressure > 90 mm Hg, or the use of antihypertensive medications), diabetes (self-reported or determined by the use of insulin or hypoglycemic medications), stroke, myocardial infarction (identified by a self-reported history of diagnosis by a physician), and an apnea hypopnea index with 4% oxygen desaturation. Additionally, baseline sleep-related covariates included the habitual sleep duration on weekdays in hours, social jetlag, defined as the actual difference between the midpoint of sleep on weekends and that on weekdays, calculated by habitual bedtime and waketime on weekdays and weekdays [25], daytime sleepiness level defined by the Epworth Sleepiness Scale [26], number of naps for five minutes or longer per week, length of sleep during naps taken on the day of assessment in hours and minutes, insomnia or poor sleep as indicated by a self-reported consumption of sleeping pills or difficulty in initiating or maintaining sleep [19], use of antidepressants or benzodiazepines, and rapid eye movement sleep percentage, which has been shown not only to negatively associate with mortality risk in community-based cohorts, including the SHHS [27], but also to be more variable than non-rapid eye-movement sleep stages across in-home PSG nights [28].

### Statistical analysis

Of the 3128 individuals analyzed, 590 (18.9%) individuals had at least one missing value in the baseline covariates. A chained equation with 20 imputed datasets was used to replace the missing data, assuming that data were missing randomly [29]. We used Cox proportional hazard models to assess associations between the individuals’ ability to obtain sufficient sleep, amount of weekend CUS, and all-cause mortality using our exposure of interest. We first assessed the individual effect of TST on mortality. Then, we assessed the joint effects of TST and CUS on mortality using the primary and secondary normal cutoffs. The secondary cutoffs were applied while excluding individuals with a PSG-measured TST between 330 and 390 min (*n* = 1100), leaving 2028 individuals for analysis. Results are shown as hazard ratios with 95% confidence intervals. To test for effect of modification in joint analysis, an interaction term between TST and CUS was entered into each model. All analyses were performed using SPSS Statistics, version 23 (IBM Japan, Tokyo). In addition to unadjusted and age/sex-adjusted models, we ran two multivariable-adjusted models. Model 1 included demographic and health covariates selected based on the known risk factors of mortality, including age, sex, race (Caucasian vs. other), body mass index, smoking status, hypertension, diabetes, apnea hypopnea index with 4% oxygen desaturation, stroke, and myocardial infarction. Model 2 further included sleep-related covariates, including the self-reported habitual sleep duration on weekdays, midsleep point on weekdays, number of daytime naps per week, length of naps, score on the Epworth Sleepiness Scale, use of antidepressants or benzodiazepines, insomnia or poor sleep, and rapid eye movement sleep percentage. Finally, we conducted sensitivity analysis by excluding those dying and censored in the first two years following baseline to exclude other possible explanations for the association of TST and CUS with mortality [30].

## Results

### Characteristics of participants

**Fig. 1:**
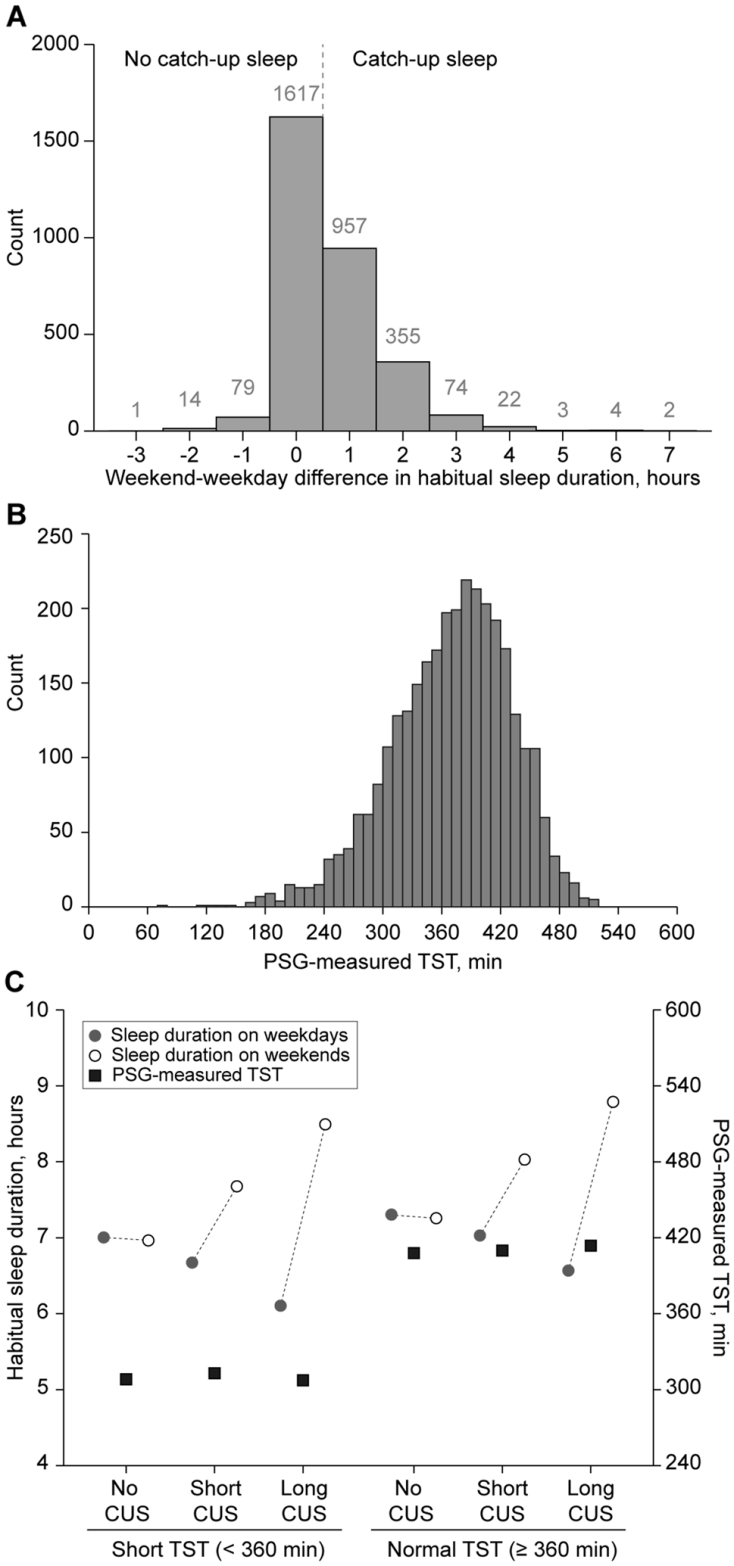
Habitual weekday and weekend sleep duration, objective sleep duration, and their relationship. **A** The distribution of weekend catch-up sleep (CUS), as represented by the difference in participants’ habitual sleep duration on weekends and that on weekdays, each self-reported in hours at baseline. **B** The distribution of participants’ total sleep time (TST) determined by in-home polysomnography at baseline. **C** The relationships between habitual weekday and weekend sleep duration, and PSG-measured TST stratified by the extent of CUS (no, short, long) and TST (short, normal)

The SHHS cohort included 3,128 middle-aged adults with a mean (standard deviation [SD]) age of 54.5 (6.6) years (40–64 years) at baseline. Participants reported sleeping habitually for 6.98 (1.13) h on weekdays and 7.59 (1.24) h on weekends. The reported habitual bedtime on weekdays and weekends were 22:55 (1:26) and 23:12 (1:32), respectively. The reported habitual waketime on weekdays and weekends were 6:11 (1:31) and 7:06 (1:26), respectively. Over half of the participants (*n* = 1713, 54.8%) did not habitually extend their sleep on weekends. Of the remaining participants who habitually caught up on their sleep on weekends (*n* = 1415, 45.2%), two-thirds (*n* = 946) reported having short (1-h) CUS, while one-third (*n* = 469) reported having long (2-h +) CUS on weekends. Participants slept for a median (interquartile range [IQR]) of 376.0 (331.0–413.5) minutes on the PSG night. The majority of participants (*n* = 1881, 60.1%) had an objectively normal sleep duration (TST ≥ 360 min), while the remaining participants (*n* = 1247, 39.9%) had an objectively short sleep duration (TST < 360 min) (Fig. 1A, B). Table 1 reports demographic, health, and sleep characteristics varying across the TST-CUS classifications. Individuals with short weekend CUS and normal sleep duration tended to have a lower body mass index, apnea hypopnea index, daytime sleepiness, fewer daytime naps, and a higher rate of rapid eye movements during sleep compared to individuals in the other CUS-TST classifications. Figure 1C illustrates the relationships between self-reported habitual sleep durations on weekdays and weekends and PSG-measured TST stratified by the TST-CUS classifications. Self-reported habitual sleep timing (i.e., bedtime, waketime) with calculated midsleep on weekdays and weekends, and self-reported frequency and length of daytime naps, stratified by the TST-CUS classifications, are shown in Supplementary Figs. S1 and S2, respectively.

**Table 1:** Baseline characteristics of participants by TST and CUS (*n* = 3128)

	Short TST (< 360 min)			Normal TST (≥ 360 min)		
Characteristic	No CUS (*n* = 688)	Short CUS (*n* = 363)	Long CUS (*n* = 196)	No CUS (*n* = 1022)	Short CUS (*n* = 583)	Long CUS (*n* = 276)
Age, mean (SD), y	56.0 (6.3)	54.5 (6.2)	53.1 (6.9)	55.2 (6.5)	52.9 (6.6)	52.1 (6.4)
Race, *n* (%)						
Caucasian	557 (81.0)	287 (79.1)	142 (72.4)	866 (84.7)	484 (83.0)	205 (74.3)
Other	131 (19.0)	76 (20.9)	54 (27.6)	156 (15.3)	99 (17.0)	71 (25.7)
Women, *n* (%)	343 (49.6)	226 (62.3)	109 (55.6)	444 (43.4)	249 (42.7)	114 (41.3)
Body mass index, mean (SD)^a^	29.1 (5.7)	28.4 (5.0)	29.9 (6.2)	28.2 (5.2)	28.0 (5.3)	28.6 (5.3)
Smoking status, *n* (%)						
Current	108 (15.7)	37 (10.2)	39 (19.9)	123 (12.0)	59 (10.1)	26 (9.4)
Former	310 (45.1)	149 (41.0)	61 (31.1)	430 (42.1)	211 (36.2)	111 (40.2)
Never	270 (39.2)	177 (48.8)	96 (49.0)	469 (45.9)	313 (53.7)	139 (50.4)
Apnea hypopnea index, mean (SD), events/h	9.8 (13.3)	10.7 (15.2)	12.0 (16.5)	8.0 (12.3)	7.7 (12.0)	8.2 (13.8)
Stroke, *n* (%)	17 (2.5)	2 (0.6)	2 (1.0)	11 (1.1)	8 (1.4)	0 (0.0)
Myocardial infarction, *n* (%)	29 (4.2)	11 (3.0)	3 (1.5)	36 (3.5)	16 (2.7)	9 (3.3)
Hypertension, *n* (%)	277 (40.3)	135 (37.2)	73 (37.2)	293 (28.7)	142 (24.4)	73 (26.4)
Diabetes, *n* (%)	46 (6.7)	21 (5.8)	12 (6.1)	38 (37.2)	27 (4.6)	18 (6.5)
Habitual sleep duration on weekdays, mean (SD)	7.01 (1.19)	6.68 (0.97)	6.12 (1.06)	7.31 (1.18)	7.03 (0.85)	6.58 (0.97)
Habitual sleep duration on weekends, mean (SD)	6.96 (1.20)	7.67 (0.98)	8.47 (1.10)	7.25 (1.19)	8.03 (0.86)	8.76 (1.04)
Weekend–weekday difference in sleep duration, mean (SD), h	–0.05 (0.29)	1.00 (0.00)	2.35 (0.98)	–0.05 (0.31)	1.00 (0.00)	2.18 (0.73)
Habitual bedtime on weekdays, mean (SD), h	22:58 (1:20)	23:00 (1:04)	23:26 (2:33)	22:46 (1:14)	22:50 (1:16)	23:03 (1:49)
Habitual waketime on weekdays, mean (SD), h	6:15 (1:24)	5:58 (1:06)	6:10 (2:34)	6:17 (1:18)	6:01 (1:03)	6:11 (2:30)
Habitual bedtime on weekends, mean (SD), h	23:14 (1:41)	23:25 (1:14)	23:24 (2:39)	23:03 (1:23)	23:13 (1:17)	23:05 (1:50)
Habitual waketime on weekends, mean (SD), h	6:43 (1:32)	7:15 (1:12)	8:16 (1:59)	6:42 (1:16)	7:16 (1:04)	8:04 (1:16)
Weekend–weekday difference in midsleep, mean (SD), h	0.38 (0.80)	0.85 (0.65)	1.77 (2.05)	0.43 (0.85)	0.89 (0.96)	1.49 (1.81)
PSG-measured TST, mean (SD), min	308.2 (42.9)	312.9 (39.4)	307.5 (41.2)	40.9 (31.2)	409.9 (33.4)	413.8 (34.7)
Stage REM sleep, mean (SD), % time	18.9 (6.8)	19.4 (6.1)	18.6 (7.1)	21.3 (5.5)	21.9 (5.3)	21.8 (5.6)
Epworth Sleepiness Scale score (0–24), mean (SD)	8.0 (4.6)	8.1 (4.3)	9.5 (4.9)	7.9 (4.4)	7.7 (4.1)	8.1 (4.6)
Number of daytime naps per week, mean (SD)	2.5 (3.4)	2.1 (2.8)	2.8 (3.8)	2.0 (3.3)	1.7 (2.6)	2.0 (2.9)
Duration of daytime naps, mean (SD), min	11.7 (29.6)	8.1 (22.7)	10.4 (40.6)	6.1 (18.9)	5.8 (18.3)	7.5 (24.7)
Antidepressant use, *n* (%)	52 (7.6)	21 (5.8)	16 (8.2)	92 (9.0)	44 (7.5)	28 (10.1)
Benzodiazepine use, *n* (%)	30 (4.4)	12 (3.3)	3 (1.5)	48 (4.7)	13 (2.2)	12 (4.3)
Insomnia or poor sleep, *n* (%)	280 (40.7)	107 (29.5)	57 (29.1)	338 (33.1)	176 (30.2)	79 (28.6)

*CUS* catch-up sleep, *PSG* polysomnography, *REM* rapid eye movement, *SD* standard deviation, *TST* total sleep time

^a^Body mass index is calculated as weight in kilograms divided by height in meters squared

### Associations of TST and CUS with survival

A total of 232 deaths (7.4%) were reported over a median (IQR) follow-up time of 12.3 (11.3–13.5) years. The regression analysis of TST showed that compared to normal sleep duration, short sleep duration was consistently associated with higher mortality (fully adjusted HR, ﻿1.45; 95% CI 1.10–1.91; Supplementary Table S1), which was also the case when the secondary cutoffs were applied (fully adjusted HR, ﻿2.01; 95% CI 1.45–2.87; Supplementary Table S1).

**Fig. 2:**
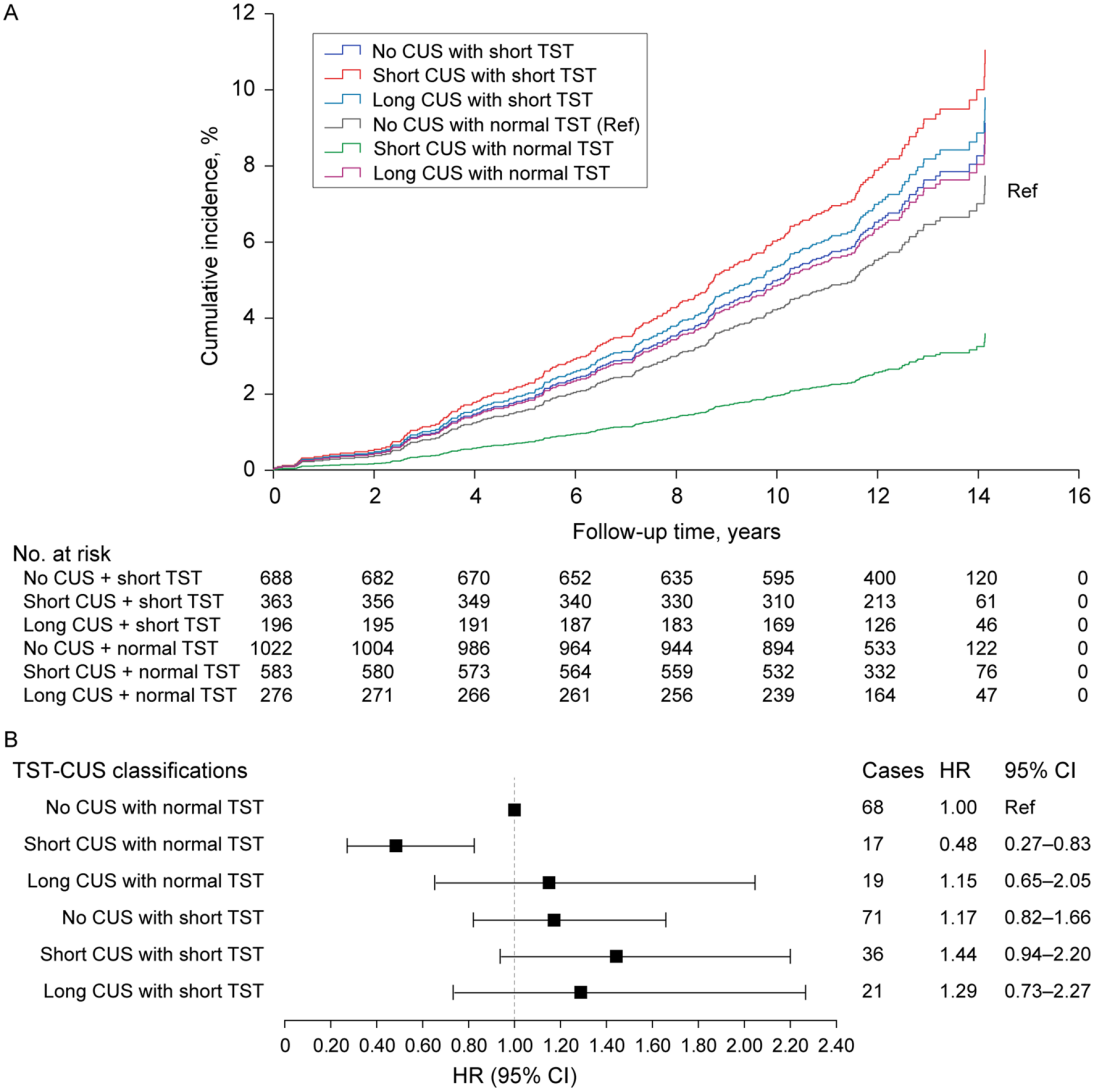
Multivariable-adjusted Cox regression plots by total sleep time (TST) and weenend catch-up sleep (CUS). Differential cumulative incidences (**A**) and hazard ratios (HRs) (**B**) from the fully adjusted Cox proportional hazard model (Model 2) are shown. *CI* confidence interval, *CUS* catch-up sleep, *Ref* reference, *TST* total sleep time

We observed a protective effect of short weekend CUS on mortality. ﻿The regression analysis of TST-CUS classifications showed that compared to no CUS with normal sleep duration (≥ 360 min), 1-h CUS with normal sleep duration was consistently associated with lower mortality, independently from health and sleep covariates (fully adjusted HR, 0.48; 95% CI 0.27–0.83; Table 2; Fig. 2A, B). When the secondary cutoffs were applied to define short (< 330 min) and normal (≥ 390 min) sleep durations, we observed both an enhanced protective effect of short weekend CUS on mortality and a newly emerged adverse effect of short weekend CUS. ﻿The regression analysis of TST-CUS classifications showed that compared to no CUS with normal sleep duration (≥ 390 min), 1-h CUS with normal sleep duration (≥ 390 min) was consistently associated with lower mortality (fully adjusted HR, 0.36; 95% CI 0.17–0.78), whereas 1-h CUS with short sleep duration (< 330 min) was consistently associated with higher mortality (fully adjusted HR, 1.84; 95% CI 1.08–3.14; Table 2)

.

**Table 2:** Mortality HRs from Cox regression of TST-CUS classifications with different TST cutoffs

		HR (95% CI)			
Predictor	Death rate (%)	Unadjusted	Age/sex-adjusted	Model 1^a^	Model 2^b^
Primary cutoff for TST (*n* = 3128)^c^					
Short TST (< 360 min)					
No CUS	71/688 (10.3)	1.52 (1.09–2.13)	1.43 (1.02–2.00)	1.21 (0.86–1.70)	1.17 (0.82–1.66)
Short CUS (1 h)	36/363 (9.9)	1.49 (0.99–2.24)	1.52 (1.01–2.29)	1.53 (1.01–2.31)	1.44 (0.94–2.20)
Long CUS (2 h or more)	21/196 (10.7)	1.51 (0.92–2.49)	1.70 (1.03–2.80)	1.43 (0.86–2.40)	1.29 (0.73–2.27)
Normal TST (≥ 360 min)					
No CUS	68/1,022 (6.7)	Ref	Ref	Ref	Ref
Short CUS (1 h)	17/583 (2.9)	0.43 (0.25–0.74)	0.50 (0.29–0.88)	0.49 (0.28–0.86)	0.48 (0.27–0.83)
Long CUS (2 h or more)	19/276 (6.9)	0.97 (0.57–1.66)	1.23 (0.72–2.11)	1.24 (0.72–2.15)	1.15 (0.65–2.05)
Secondary cutoffs for TST (*n* = 2028)^d^					
Short TST (< 330 min)					
No CUS	51/425 (12.0)	1.97 (1.30–2.99)	1.84 (1.21–2.80)	1.49 (0.97–2.30)	1.51 (0.96–2.37)
Short CUS (1 h)	25/211 (11.8)	2.04 (1.23–3.37)	2.04 (1.23–3.39)	2.05 (1.23–3.41)	1.84 (1.08–3.14)
Long CUS (2 h or more)	15/126 (11.9)	1.84 (1.00–3.38)	2.08 (1.13–3.84)	1.80 (0.95–3.40)	1.47 (0.73–2.98)
Normal TST (≥ 390 min)					
No CUS	40/683 (5.9)	Ref	Ref	Ref	Ref
Short CUS (1 h)	9/390 (2.3)	0.36 (0.17–0.77)	0.42 (0.20–0.91)	0.39 (0.18–0.84)	0.36 (0.17–0.78)
Long CUS (2 h or more)	10/193 (5.2)	0.81 (0.39–1.69)	1.03 (0.50–2.16)	1.04 (0.48–2.22)	0.87 (0.39–1.96)

*CI* confidence interval, *CUS* catch-up sleep, *HR* hazard ratio, *Ref* reference, *TST* total sleep time

^a^Model 1 included age, sex, race (Caucasian vs. other), body mass index, smoking status, hypertension, diabetes, apnea hypopnea index with 4% oxygen desaturation, stroke, and myocardial infarction

^b^Model 2 included Model 1 plus the self-reported habitual sleep duration on weekdays, difference in midsleep between weekends and weekdays (social jetlag), number of daytime naps per week, duration of naps, score on the Epworth Sleepiness Scale, use of antidepressants or benzodiazepines, insomnia or poor sleep, and rapid eye-movement sleep percentage

^c^*P* = 0.67 for interaction between continuous TST and CUS variables

^d^*P* = 0.63 for interaction between continuous TST and CUS variables

The sensitivity analysis, including those who survived the first two years from baseline, did not show any different results, except for an increased mortality risk of no CUS with short sleep duration (< 330 min) defined by a secondary cutoff (Supplementary Table S2).

## Discussion

In this secondary analysis of a community-based, prospective cohort study, we found both beneficial and adverse health effects of weekend CUS that differed depending on the amount of CUS and the ability to obtain sufficient sleep among middle-aged adults. If one slept for a normal duration (≥ 360 min) on the PSG night, a habitual short (1-h) weekend CUS was associated with a lower mortality risk, compared to no weekend CUS. When stricter cutoffs were applied to define short (< 330 min) and normal (≥ 390 min) sleep durations, the harmful effect of short (1-h) weekend CUS newly emerged, whereas the protective effect of short (1-h) weekend CUS was strengthened.

Our findings may help clarify which individuals would benefit more from weekend CUS. The link between weekend CUS and lower mortality was observed only among individuals with objectively normal sleep duration (as either ≥ 360 min or ≥ 390 min). The protective effect of 1-h CUS, as compared to no CUS, if sleep duration is normal, implies that middle-aged adults potentially carry some amount of sleep debt despite their normal ability to obtain sufficient sleep, but a slight amount of weekend CUS could successfully compensate for their sleep debt as far as they maintain that sleep ability. This finding partly aligns with the reported beneficial effects of weekend CUS [22, 23, 31] and newly suggest that only a very small fraction of additional wakefulness is allowed to maintain against biological sleep needs during weekdays so that weekend CUS could efficiently liquidate its cumulative cost.

Notwithstanding, no protective effect was seen for a high amount (2-h or more) of weekend CUS with normal sleep duration. Health and sleep covariates, including social jetlag and daytime napping, did not influence this null finding. The presence of such a high amount of CUS itself may represent the accumulated sleep debt that is not substantially compensated for during weekends, even when their ability to obtain sufficient sleep is not objectively impaired. A high amount of weekend CUS, compared to a low amount, is more likely to put individuals at risk of circadian misalignment, typically due to delayed sleep offset on weekends (work-free days). Given that social jetlag mainly represents actual sleep timing as a measure for circadian misalignment, it could be substantially influenced by sleep loss [25]; this finding suggests that the individual effect of accumulated sleep debt counteracts the protective effects of weekend CUS on mortality, independently from sleep timing effects. Therefore, it is plausible that 2-h + CUS failed to exert a protective effect against mortality, as compared to no CUS. In contrast, the protective effect of short (1-h) weekend CUS with normal sleep duration remained robust after accounting for social jetlag, suggesting the individual effect of the amount of weekend CUS itself on mortality, independent of the circadian drift of sleep phase across the weekday–weekend cycle.

The lack of the protective effect of weekend CUS on mortality among individuals with objectively short sleep duration (as either < 360 min or < 330 min) suggests that when a weekday sleep amount is substantially reduced, the accumulated sleep debt greatly exceeds the homeostatic, restorative potential of CUS for only two weekend nights. Interestingly, when short sleep duration was defined by a second, stricter definition (< 330 min), the harmful effect of weekend CUS remained significant after adjustments among individuals with short (1-h) and not long (2-h +) weekend CUS. Additionally, a sensitivity analysis showed a harmful effect of no weekend CUS, as well as short weekend CUS, with short sleep duration (< 330 min). These findings appear to align with experimental evidence of the harmful effects of sleep debt due to short weekday sleep (e.g., sleep restriction to 4 or 5 h per night for weekdays) on metabolic and endocrine function, which require more than two days to recover [6, 7, 32]. On the other hand, long (2-h +) weekend CUS with an objectively short sleep duration was not associated with an increased mortality risk, as compared to no CUS with objectively normal sleep duration. This finding suggests that a high amount of CUS could help stave off the harmful effects of accumulated sleep debt related to the impaired ability to obtain sufficient sleep. A large-scale longitudinal epidemiological study also suggested that weekend medium or long sleep mitigates the harmful effects related to weekday short sleep on mortality [4]. However, this previous sudy did not differentiate the effects of varying degrees of weekend CUS on mortality among individuals with weekday short sleep. Overcoming this limitation, our present study implies that long, but not short, weekend CUS could mitigate the harmful effects of potential sleep debt among those with weekday short sleep. Moreover, the harmful effect of no weekend CUS with short sleep duration, compared to no CUS with normal sleep duration, was only observed in the sensitivity analysis with a stricter cutoff to dfine short sleep duration (< 330 min). A possible explanation for this is that a certain proportion of individuals with no CUS with objectively short sleep duration were genetically resistant to sleep loss, as represented by short sleepers [33, 34]. In addition, the circadian stability of habitual sleep timing related to no weekend CUS could also alleviate the adverse health effects of short sleep duration [3].

The strengths of our study include its relatively large size, its prospective design, the availability of objective sleep measures, including those that are known to affect mortality (e.g., sleep duration, as well as REM sleep and apnea hypopnea index), and the sensitivity analysis conducted to control for some of the reverse causality. However, this study also has several limitations. A single-night PSG study could underestimate sleep duration due to the first-night effect. However, a study using data from the SHHS, in which some of the SHHS participants underwent two PSG recordings, found no significant differences in sleep duration between the two nights [28]. Therefore, our findings could not be fully explained by the first-night effect. Nonetheless, we cannot be sure that the obtained PSG-measured and self-reported sleep durations are truly representative of their habitual weekday and weekend sleep durations, because these sleep durations were assessed only at baseline, and because exact information was unavailable about whether participants underwent PSG on a workday even though most of them started the PSG on a weekday. In relation to this issue, another limitation is that the interpretation of our results could be speculative as we relied on self-reported data to assess the extent of their habitual weekend CUS. Therefore, our findings need to be confirmed through future studies that associate the objective difference in habitual sleep durations between weekdays and weekends and its changes over time with health outcomes, for instance, using wearable sleep technology. Moreover, while we adjusted for the confounding effects of sleep apnea and insomnia symptoms, other sleep disorders might have influenced our results. However, our findings suggest that individuals who maintain their sleep ability and perceive that their sleep habits need weekend CUS, but its small amount may benefit more from weekend CUS. Furthermore, from a longitudinal perspective, once their ability to obtain sufficient sleep becomes impaired by, for instance, aging or certain pathologies, this small amount of weekend CUS will not function properly and could, in turn, deteriorate health outcomes. An objective assessment of sleep habits may help identify the habit of weekend CUS that requires caution. With the development of wearable sleep technology, more reliable and valid sleep assessment methods will become available nationally. Meanwhile, people’s sleep debt is difficult to solve in modern society. Under such circumstances, our findings will be important in minimizing the negative effects of sleep debt and considering appropriate sleep habits promoting public health.

## Conclusions

In a prospective community-based cohort that followed up 3,128 middle-aged adults (40–64 years) for 12 years for mortality outcomes, we addressed the longitudinal association of the habit of weekend CUS and the ability to obtain sufficient sleep with all-cause mortality. We revealed that individuals who reported extending their sleep on weekends by only a short period of time (1 h) had a lower mortality risk, compared to those who reported not extending their sleep on weekends, if they slept for a normal duration (≥ 360 min) on baseline PSG, whereas no protective effect was found among those who slept for a short duration (< 360 min). When objectively short and normal sleep durations were more strictly defined, whereas the protective effect of short weekend CUS on mortality became more obvious among those with objectively normal sleep duration, this short weekend CUS was associated with a higher mortality risk among those with an objectively short sleep duration. There was no increase in the mortality risk among those who reported extending their sleep on weekends by a long period of time (2 h or more), even when they only slept for a short duration on PSG. Our results emphasize the importance of balancing between the extent of CUS required to compensate for sleep debt that accumulates during weekdays and one’s ability to obtain sufficient sleep that could minimize the accumulation of nightly sleep loss among middle-aged adults. Weekend CUS may substantially benefit individuals who maintain their sleep ability and thus require a small amount of CUS. Further studies are needed to confirm these findings and examine underlying mechanisms.

### Supplementary Information

Below is the link to the electronic supplementary material.

Supplementary file1 (DOCX 200 KB)
