# Supplementary material for: A prospective study of the association of weekend catch-up sleep and sleep duration with mortality in middle-aged adults
Source: Sleep Biol Rhythms. 2023 May 5;21(4):409–18. doi: 10.1007/s41105-023-00460-6 (PMC10900010; doi:10.1007/s41105-023-00460-6)
Supplement: Supplementary file 1 — Supplementary file1 (DOCX 200 KB) [file 41105_2023_460_MOESM1_ESM.docx]

**Supplementary Material**

**A prospective study of the association of weekend catch-up sleep and sleep duration with mortality in middle-aged adults**

Takuya Yoshiike, Aoi Kawamura, Tomohiro Utsumi, Kentaro Matsui, Kenichi Kuriyama

**Figure S1.** Self-reported sleep timing on weekdays and weekends by TST-CUS classifications

**Figure S2.** Self-reported daytime naps by TST-CUS classifications

**Table S1.** Mortality HRs from Cox regression of TST classifications with different TST cutoffs

**Table S2.** Sensitivity analyses of mortality HRs from Cox regression of TST-CUS classifications for participants who survived first two years of study

**Figure S1. Self-reported sleep timing on weekdays and weekends by TST-CUS classifications**


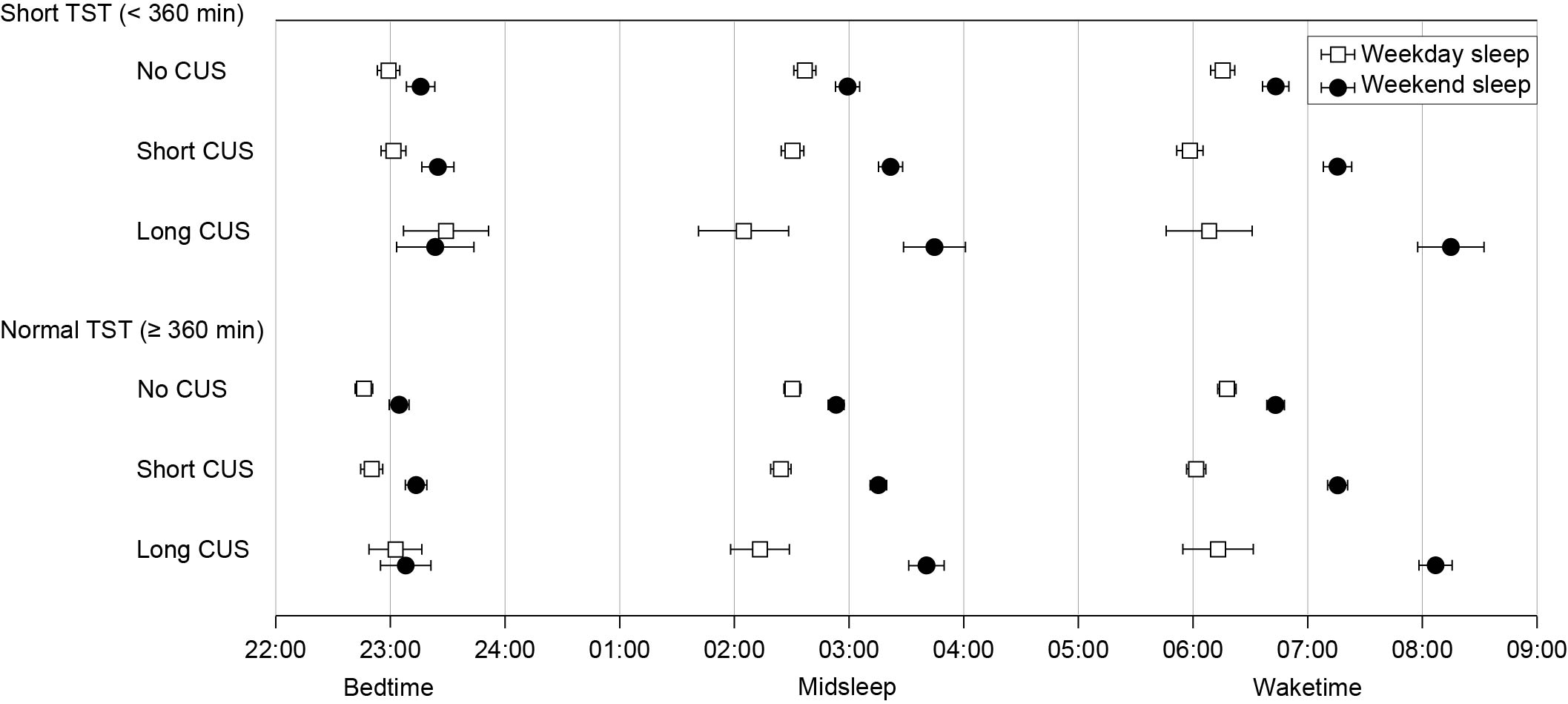


Means and 95% confidence intervals are shown for bedtime, midsleep, and waketime on weekdays and weekends.

CUS, catch-up sleep; TST, total sleep time.

**Figure S2.** **Self-reported daytime naps by TST-CUS classifications**

**
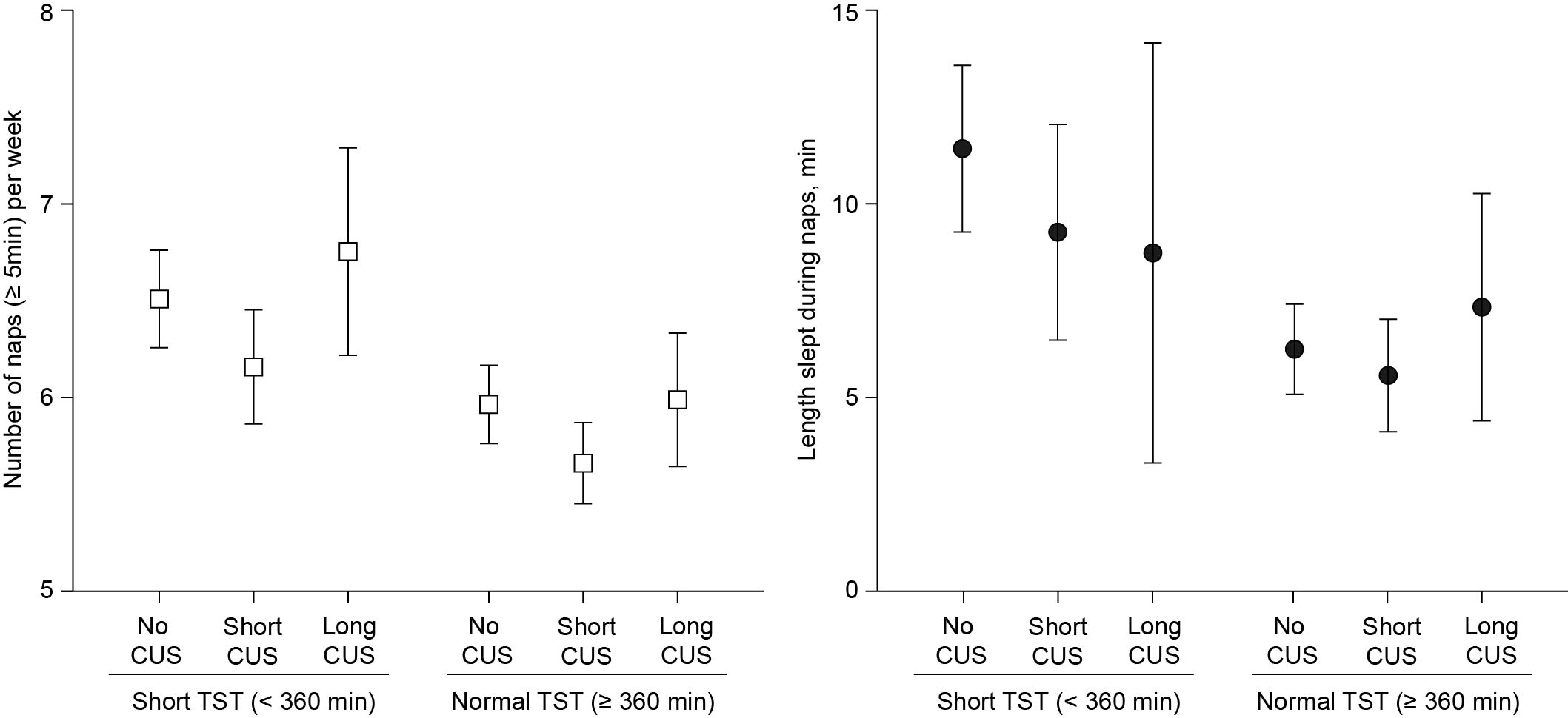
**

Means and 95% confidence intervals are shown for the self-reported frequency (left) and length of daytime naps (right).

CUS, catch-up sleep; TST, total sleep time.

**Table S1. Mortality HRs from Cox regression of TST classifications**

|  |  | **HR (95% CI)** | | | |
| --- | --- | --- | --- | --- | --- |
| **Predictor** | **Death rate (%)** | **Unadjusted** | **Age/sex-adjusted** | **Model 1^a^** | **Model 2^b^** |
| **Primary cutoff for TST (n = 3,128)** |  |  |  |  |  |
| Short TST (< 360 min) | 129/1247 (10.3) | 1.85 (1.43–2.40) | 1.68 (1.30–2.19) | 1.50 (1.15–1.96) | 1.45 (1.10–1.91) |
| Normal TST (≥ 360 min) | 103/1881 (5.5) | Ref | Ref | Ref | Ref |
| **Secondary cutoffs for TST (n = 2,028)** |  |  |  |  |  |
| Short TST (< 330 min) | 91/762 (11.9) | 2.56 (1.84–3.56) | 2.31 (1.65–3.22) | 2.04 (1.45–2.87) | 2.01 (1.45–2.87) |
| Normal TST (≥ 390 min) | 58/1266 (4.6) | Ref | Ref | Ref | Ref |

^a^ Model 1 included age, sex, race (Caucasian vs. other), body mass index, smoking status, hypertension, diabetes, apnea hypopnea index with 4% oxygen desaturation, stroke, and myocardial infarction.

^b^ Model 2 included Model 1 plus the self-reported habitual sleep duration on weekdays, midpoint of sleep on weekdays, number of daytime naps per week, duration of naps, score on the Epworth Sleepiness Scale, use of antidepressants or benzodiazepines, insomnia or poor sleep, and rapid eye-movement sleep percentage.

CI, confidence interval; HR, hazard ratio; Ref, reference; TST, total sleep time.

**Table S2. Sensitivity analyses of mortality HRs from Cox regression of TST-CUS classifications for participants who survived first two years of study**

|  |  | **HR (95% CI)** | | | |
| --- | --- | --- | --- | --- | --- |
| **Predictor** | **Death rate (%)** | **Unadjusted** | **Age/sex-adjusted** | **Model 1^a^** | **Model 2^b^** |
| **Primary cutoff for TST (n = 3,083)** |  |  |  |  |  |
| Short TST (< 360 min) |  |  |  |  |  |
| No CUS | 68/681 (10.0) | 1.61 (1.14–2.28) | 1.51 (1.07–2.14) | 1.29 (0.90–1.83) | 1.17 (0.82–1.66) |
| Short CUS (1h) | 34/356 (9.6) | 1.51 (0.98–2.31) | 1.56 (1.01–2.40) | 1.56 (1.01–2.40) | 1.44 (0.94–2.20) |
| Long CUS (2h or more) | 21/196 (10.7) | 1.67 (1.01–2.76) | 1.89 (1.14–3.15) | 1.59 (0.95–2.68) | 1.29 (0.73–2.27) |
| Normal TST (≥ 360 min) |  |  |  |  |  |
| No CUS | 61/1004 (6.1) | Ref | Ref | Ref | Ref |
| Short CUS (1h) | 17/577 (2.9) | 0.46 (0.27–0.81) | 0.55 (0.32–0.96) | 0.54 (0.31–0.95) | 0.48 (0.27–0.83) |
| Long CUS (2h or more) | 16/269 (5.9) | 0.94 (0.53–1.66) | 1.20 (0.67–2.14) | 1.21 (0.67–2.19) | 1.15 (0.65–2.05) |
| **Secondary cutoffs for TST (n = 1,988)** |  |  |  |  |  |
| Short TST (< 330 min) |  |  |  |  |  |
| No CUS | 50/423 (11.8) | 2.10 (1.37–3.23) | 1.97 (1.28–3.03) | 1.58 (1.02–2.47) | 1.61 (1.01–2.56) |
| Short CUS (1h) | 22/206 (10.7) | 1.98 (1.16–3.36) | 1.97 (1.15–3.36) | 1.97 (1.15–3.38) | 1.75 (1.00–3.07) |
| Long CUS (2h or more) | 15/126 (11.9) | 2.01 (1.09–3.74) | 2.26 (1.21–4.20) | 1.92 (1.01–3.64) | 1.51 (0.74–3.09) |
| Normal TST (≥ 390 min) |  |  |  |  |  |
| No CUS | 37/670 (5.5) | Ref | Ref | Ref | Ref |
| Short CUS (1h) | 9/386 (2.3) | 0.37 (0.17–0.79) | 0.45 (0.21–0.91) | 0.43 (0.20–0.93) | 0.38 (0.18–0.84) |
| Long CUS (2h or more) | 8/187 (4.3) | 0.67 (0.30**–**1.51) | 0.88 (0.39**–**2.16) | 0.89 (0.38**–**2.08) | 0.72 (0.29**–**1.78) |

^a^ Model 1 included age, sex, race (Caucasian vs. other), body mass index, smoking status, hypertension, diabetes, apnea hypopnea index with 4% oxygen desaturation, stroke, and myocardial infarction.

^b^ Model 2 included Model 1 plus the self-reported habitual sleep duration on weekdays, difference in midsleep between weekends and weekdays (social jetlag), number of daytime naps per week, duration of naps, score on the Epworth Sleepiness Scale, use of antidepressants or benzodiazepines, insomnia or poor sleep, and rapid eye-movement sleep percentage.

CI, confidence interval; CUS, catch-up sleep; HR, hazard ratio; Ref, reference; TST, total sleep time.
